# Specialization of Home Health Agencies to Deliver Care for Medicare Advantage Patients

**DOI:** 10.1001/jamanetworkopen.2025.25336

**Published:** 2025-08-04

**Authors:** Amanda C. Chen, Christina X. Fu, David C. Grabowski

**Affiliations:** 1 Department of Health Care Policy, Harvard Medical School, Boston, Massachusetts; 2 Harvard Graduate School of Arts and Sciences, Boston, Massachusetts

## Abstract

**Importance:**

Enrollment in Medicare Advantage (MA) is expected to continue growing. Previous studies have examined differences in the use and quality of home health care between MA and traditional Medicare, but less is known about outcomes among patients receiving care from agencies with greater exposure to MA patients.

**Objective:**

To examine the association between home health agency (HHA) experience with caring for MA patients and quality of care delivered.

**Design, Setting, Participants:**

This cohort study included patients continuously enrolled in MA and who received HHA care in 2019. The data analysis was performed between July 16, 2024, and January 16, 2025.

**Exposure:**

Medicare beneficiaries who received home health care from agencies with differing levels of MA specialization.

**Main Outcomes and Measures:**

Primary outcomes included hospitalizations during the HHA episode and after HHA discharge (at 30 and 90 days), length of stay, and total number of visits. Secondary outcomes included postdischarge mortality and nursing home admission. The outcomes were measured using instrumental variable analysis. The treatment variable was a continuous measure of the HHA-level share of MA patients. The instrumental variable was the differential distance from the nearest MA-specialized HHA to nearest non–MA-specialized HHA (based on the 75th percentile of the HHA-level share of MA patients from January 1 to December 31, 2019 [ie, ≥36.4%]).

**Results:**

The study included 749 719 MA patients who received HHA care in 2019 (mean [SD] age, 76.2 [10.4] years; 61.6% female; 26.3% with dual eligibility), of whom 65.4% received care from an MA-specialized HHA and 34.6% received care from a non–MA-specialized HHA. A 1-mile increase in differential distance was associated with a lower likelihood of admission to more MA-specialized HHAs (0.3 percentage points; SE, 0.015 percentage points; *F* statistic, 450.73). In the instrumental variable analysis, receiving care from more specialized HHAs was associated with a shorter length of stay (coefficient [SE], −15.14 [2.84] days) and fewer total HHA visits (coefficient [SE], −9.40 [1.15] visits) alongside more hospitalizations and nursing home admissions after discharge from the HHA.

**Conclusion and Relevance:**

In this cohort study of MA patients who received HHA care, those receiving care from more MA-specialized HHAs had lower service use during the HHA episode, but no clear differences compared with non–MA-specialized HHAs were observed in care use after discharge. These findings are important given the costs associated with delivering HHA care and the expected growth in MA enrollment.

## Key Points

**Question:**

What is the association between home health agency (HHA) specialization in delivering care for Medicare Advantage (MA) patients and quality of care received?

**Findings:**

In this cohort study of 749 719 patients enrolled in MA who received HHA care in 2019, those receiving care from MA-specialized HHAs had fewer visits and shorter lengths of stay. More hospitalizations and nursing home admissions after discharge from the HHA, but no difference in mortality, were observed.

**Meaning:**

These findings suggest that although MA-specialized HHAs provide fewer services with no negative association with patient outcomes during the HHA episode, there are unclear implications for service use after discharge.

## Teaser

This cohort study examines the association between home health agencies specializing in delivering care to patients with Medicare Advantage and quality of care received.

## Introduction

In 2023, US national spending for home health care increased more than 10% from the prior year, totaling nearly $150 billion.^1^ Use of home health is expected to continue to grow because of the aging population and increased preference for receiving care at home instead of within institutional care settings since the COVID-19 pandemic.^2^ Understanding the role of Medicare Advantage (MA) in covering health care services, including home health care, is also increasingly important given signals by the Trump administration to accelerate enrollment in managed care plans, such as MA.^3^

Prior studies have compared outcomes between patients enrolled in MA and traditional Medicare, given differences in MA’s use management to reduce costs for prior authorizations, beneficiary cost-sharing, and limited networks. These studies found that MA beneficiaries are less likely to receive care from home health agencies (HHAs)^4,5,6^ and conditional on receiving care, are less likely to receive care from high-quality HHAs,^7^ have shorter lengths of stay,^8,9^ and receive fewer visits^9,10^ compared with those enrolled in traditional Medicare.

Our study examines the role of MA in delivering home health care by focusing on beneficiaries enrolled in MA plans exclusively. We hypothesized that HHAs that have greater exposure or experience with delivering care for MA patients may have more efficient processes and be better able to address some common burdens, such as authorization limits, associated with delivering care for MA patients. As a result, patients admitted to these MA-specialized HHAs may have better outcomes than patients admitted to HHAs that might have less experience (non–MA-specialized HHAs).

## Methods

This cohort study was deemed not to be human participants research by the Harvard Medical School Institutional Review Board; therefore, it was exempt from further review and informed consent. The study followed the Strengthening the Reporting of Observational Studies in Epidemiology (STROBE) reporting guideline.

A 100% sample was obtained from the Medicare Beneficiary Summary File (MBSF),^11^ Medicare Provider Analysis and Review (MedPAR) file,^12^ Medicare Encounter HHA claims,^13^ and the Outcome and Assessment Information Set (OASIS) from January 1 to December 31, 2019.^14^ We also used 2020 data to follow HHA episodes that started at the end of 2019. We obtained HHA characteristics from the Centers for Medicare & Medicaid Services HHA Care Compare (2019)^15^ and used the Minimum Data Set, version 3.0 (2019) to identify nursing home admissions.^16^

### Sample

The sample was representative of patients who were continuously enrolled in MA during the calendar year, resided in the contiguous 48 states, and received care from an HHA in 2019 (eFigure in Supplement 1). For each patient, we defined their HHA episode based on start-of-care and discharge dates recorded in OASIS and excluded those who had an OASIS record but were missing corresponding HHA claims (eTable 8 in Supplement 1). We excluded patients who may have used home health in 2018 and focused on the first episode among those with recurrent HHA episodes in 2019.

### Covariates

Covariates derived from the MBSF included age, sex, race and ethnicity (Black, Hispanic, White, or other [including American Indian or Alaska Native, Asian, and unknown]) and dual-eligibility status. Race and ethnicity are important patient demographic characteristics commonly controlled for in studies of HHA care. We used MedPAR to define postacute care patients as those discharged from an acute care hospital with a discharge destination of home health care and who subsequently initiated HHA care within 14 days. Clinical characteristics from OASIS included patients’ functional abilities (cognition, self-care, mobility, and stairs) prior to initiating HHA care and severity rating of their primary diagnosis. Home health agency characteristics included ownership (nonprofit, for-profit, or government owned), star rating (1-5 stars), and location (urban, suburban, or rural). We also used HHA claims to calculate the total number of patients receiving care from each HHA.

### Outcomes

Our primary hospitalization measures from MedPAR captured any hospitalization during the HHA episode and hospitalizations within 30 and 90 days after discharge from the HHA. We also measured total length of stay in the HHA based on admission and discharge dates and the total number of visits received during the HHA episode using revenue center codes from claims. Total visits included the number of skilled nursing, physical therapy, occupational therapy, speech-language pathology, medical social services, and home health aide visits. Secondary outcomes included mortality based on the MBSF (30 and 90 days from HHA discharge); total visits by skilled nursing, therapy (physical therapy, occupation therapy, and/or speech-language pathology), medical social services, and home health aide visits separately; nursing home admission using the Minimum Data Set (at 30, 90, and 180 days); and conversion to long-stay status (at 90, 180, and 365 days), defined as a nursing home stay of at least 90 days.

### Statistical Analysis

To account for patient selection into HHAs, we took an instrumental variable approach using 2-stage least squares. We used this approach because the treatment variable, admission to an MA-specialized HHA, is endogenous and may lead to biased estimates when using a naive ordinary least-squares approach. The endogeneity we addressed relates to unobserved patient-level characteristics that may be correlated with both receiving care at an MA-specialized HHA and our outcomes of interest. For example, a beneficiary with greater family support may be more informed in choosing an HHA and have more help at home to avoid hospitalization. Additionally, MA specialization may be correlated with agency-level factors, such as management, operations, and quality, and population-level attributes, which contribute to differences in the willingness to care for greater shares of MA patients and lead to differences in patient outcomes. Thus, there may be endogeneity at both the patient level and agency level. Our approach accounts for patient-level differences and controls for measured HHA characteristics, but there may be unmeasured HHA-level characteristics that are correlated with the outcome measures and that bias our results because they are unaccounted for in our approach.

**Figure 1.**
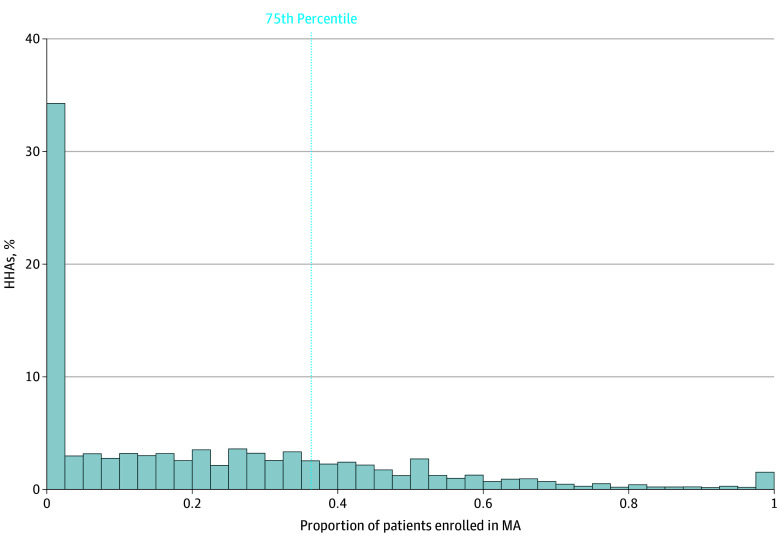
Home Health Agency (HHA)–Level Distribution of Medicare Advantage (MA) Patients The vertical dotted line indicates the HHA-level share of MA patients in 2019.

An instrumental variable approach approximates the random assignment of patients to treatment. Our treatment variable was a continuous measure of the HHA-level share of MA patients in 2019 (Figure 1). We also used this measure to determine the cutoff for MA specialization when creating our instrument, differential distance, based on the 75th percentile of the distribution (ie, ≥36.4% of the HHA’s Medicare patients enrolled in MA). Our instrument measured the differential distance (in miles) from the nearest HHA that specialized in delivering care to MA patients (MA-specialized HHA) to the nearest HHA that was not (non–MA-specialized HHA) and was calculated using the centroid of the patient’s zip code to latitude and longitude coordinates for the HHA home office location. Although HHA care is delivered at a patient’s home rather than in an institutional setting, distance to the HHA home office has been used in prior instrumental variable studies and is conceptually relevant for several reasons.^17,18^ First, the home office location is an important marker of patients’ potential exposure to HHA care as distance may influence their choice of an agency, including when searching for HHAs on the Centers for Medicare & Medicaid Services Care Compare website to identify nearby agencies.^19^ Second, HHAs may provide services to patients based on their proximity to the home office location due to the availability and travel time of their staff, who may need to pick up supplies from the home office.

The adjusted outcomes from the instrumental variable analysis represent the outcome for the marginal patient, that is, a patient who received care from an MA-specialized HHA because of the relative distance between the nearest MA-specialized and non–MA-specialized HHA. All regression models controlled for the patient and HHA characteristics, as well as county fixed effects with standard errors clustered at the HHA level. In supplemental analyses, we tested specifications using an MA specialization cutoff based on the 90th percentile (55.6%) and a binary measure of MA specialization instead of a continuous measure.

There is a series of assumptions that must be met for our instrumental variable analysis to be valid in addressing patient-level endogeneity. First is relevance, that is, as the distance to the nearest MA-specialized HHA increases relative to the distance to the nearest non–MA-specialized HHA, patients should be less likely to receive care from an MA-specialized HHA. We tested this assumption in the first-stage regression in which a large *F* statistic suggests a strong association between our instrument and receiving care from an MA-specialized HHA. Second is the exclusion restriction, which assumes that the instrument only affects the outcome through the treatment variable. This assumption would be violated if individuals chose their HHA based on knowledge about the share of MA patients cared for by each HHA. As a check, we conducted a falsification test for individuals who receive care from an HHA with a home office that is located far from their primary residence (eg, 50, 100, or 150 miles away). Third is independence between the instrument and unobserved patient-level confounders. We evaluated the plausibility of this assumption by comparing observable patient characteristics to check whether differential distance is independent of the set of observed characteristics. If so, our confidence would increase that the instrument is also independent of other, unobserved patient-level factors.

All analyses were conducted using SAS, version 9.4 (SAS Institute Inc) and Stata, version 17 (StataCorp LLC). A 2-sided *P* < .05 was considered statistically significant.

## Results

The study included 749 719 patients who were enrolled in MA and received HHA care in 2019 (mean [SD] age, 76.2 [10.4] years; 61.6% female and 38.4% male; 14.6% of Black, 8.1% of Hispanic, 74.3% of White, and 2.4% of other race and ethnicity) (Table 1). The percentage of patients with dual eligibility was 26.3%, and 36.3% received postacute HHA care. The percentage of HHAs that were for-profit was 54.1%, with a mean (SD) star rating of 3.5 (0.8) stars, and 65.7% were in urban areas. The percentage of patients who received care from an MA-specialized HHA was 65.4%, whereas 34.6% received care from a non–MA-specialized HHA.

**Table 1.** Characteristics of the Study Sample

Characteristic	MA patients, No. (%)			
	Overall (N = 749 719)	Non–MA-specialized HHA (n = 259 301)		MA-specialized HHA (n = 490 418)
**Patient**				
Age, mean (SD), y	76.2 (10.4)	76.1 (10.4)		76.2 (10.4)
Sex				
Female	7461 796 (61.6)	158 953 (61.3)		302 843 (61.8)
Male	287 923 (38.4)	100 348 (38.7)		187 575 (38.2)
Race and ethnicity				
Black	109 324 (14.6)	38 900 (15.0)		70 424 (14.4)
Hispanic	60 497 (8.1)	14 860 (5.7)		45 637 (9.3)
White	557 071 (74.3)	198 086 (76.4)		358 985 (73.2)
Other^a^	17 874 (2.4)	5792 (2.2)		12 082 (2.5)
Dual eligibility	196 825 (26.3)	65 813 (25.4)		131 012 (26.7)
Postacute care	272 013 (36.3)	93 751 (36.2)		178 262 (36.4)
Prior functioning				
Cognitive status				
Dependent	20 124 (2.7)	7568 (2.9)		12 556 (2.6)
Needed some help	119 110 (15.9)	42 995 (16.6)		76 115 (15.5)
Independent	227 200 (30.3)	76 621 (29.6)		150 579 (30.7)
Missing	382 404 (51.0)	131 833 (50.8)		250 571 (51.1)
Unknown	881 (0.1)	284 (0.1)		597 (0.1)
Mobility				
Dependent	9966 (1.3)	3575 (1.4)		6391 (1.3)
Needed some help	121 010 (16.1)	43 442 (16.8)		77 568 (15.8)
Independent	233 745 (31.2)	79 650 (30.7)		154 095 (31.4)
Missing	382 294 (51.0)	131 807 (50.8)		250 587 (51.1)
Unknown	2604 (0.4)	827 (0.3)		1777 (0.4)
Self-care				
Dependent	11 898 (1.6)	4228 (1.63%)		7670 (1.6)
Needed some help	131 830 (17.6)	47 049 (18.2)		84 781 (17.3)
Independent	223 256 (29.8)	76 086 (29.3)		147 170 (30.0)
Missing	382 392 (51.0)	131 821 (50.8)		250 571 (51.1)
Unknown	343 (0.1)	117 (0.1)		226 (0.1)
Stairs				
Dependent	17 695 (2.4)	6381 (2.5)		11 314 (2.3)
Needed some help	125 601 (16.8)	45 930 (17.7)		79 671 (16.3)
Independent	181 536 (24.2)	62 287 (24.0)		119 249 (24.3)
Missing	384 482 (51.3)	132 542 (51.1)		251 940 (51.4)
Unknown	40 405 (5.4)	12 161 (4.7)		28 244 (5.8)
Primary diagnosis severity rating				
Asymptomatic, no treatment needed at this time	68 (<0.1)	17 (<0.1)		51 (<0.1)
Symptoms well controlled with current therapy	4784 (0.6)	979 (0.4)		3805 (0.9)
Symptoms controlled with difficulty, affecting daily functioning	101 459 (13.5)	32 548 (12.6)		68 911 (14.1)
Symptoms poorly controlled, patient needs frequent adjustment in treatment and dose monitoring	187 981 (25.1)	70 788 (27.3)		117 193 (23.9)
Symptoms poorly controlled, history of rehospitalizations	24 329 (3.3)	8805 (3.4)		15 524 (3.2)
Missing	431 098 (57.5)	146 464 (56.4)		284 934 (58.1)
**HHA**				
Ownership				
Nonprofit	213 371 (28.5)	68 561 (26.4)		144 810 (29.5)
For-profit	405 701 (54.1)	160 603 (61.9)		245 098 (50.0)
Government	130 647 (17.4)	30 137 (11.6)		100 510 (20.5)
Star rating, mean (SD), stars	3.5 (0.8)	3.7 (0.7)		3.4 (0.8)
Location				
Urban	492 642 (65.7)	160 415 (61.9)		332 227 (67.7)
Suburban	249 473 (33.3)	92 683 (35.7)		156 790 (32.0)
Rural	7604 (1.0)	6203 (2.4)		1401 (0.3)
Annual No. of patients, mean (SD)	561.7 (708.6)	429.3 (471.8)		631.7 (797.3)
**Outcomes**				
Hospitalization during HHA episode	58 823 (7.9)	21 422 (8.3)	37 401 (7.6)	
Hospitalizations after discharge				
30 d	58 900 (7.9)	20 733 (8.0)	38 167 (7.8)	
90 d	115 672 (15.4)	40 643 (15.7)	75 029 (15.3)	
Length of stay, mean (SD), d	38.1 (36.6)	41.2 (38.8)	36.4 (35.3)	
Total visits, mean (SD), No.	10.8 (8.9)	11.8 (9.3)	10.2 (8.6)	

Abbreviations: HHA, home health agency; MA, Medicare Advantage.

^a^ Includes American Indian or Alaska Native, Asian, and unknown. Categories combined due to small sample sizes.

**Figure 2.**
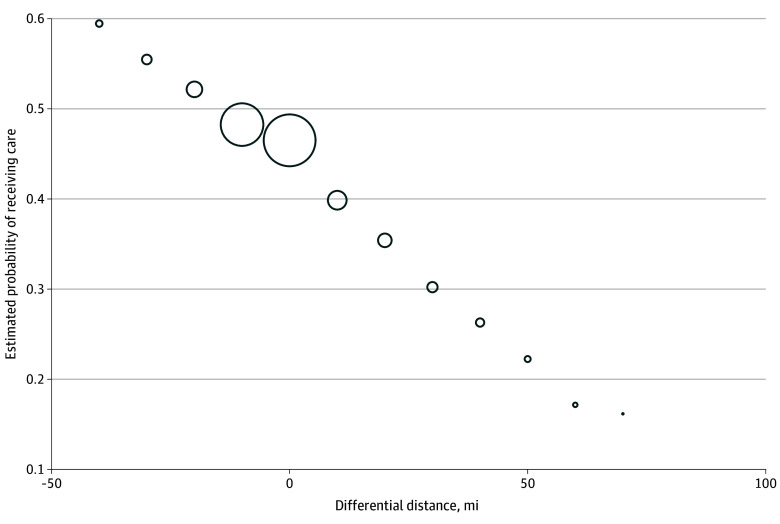
First-Stage Regression Results The size of the bubble represents the relative sample size at each measure of differential distance rounded to the nearest 10 miles. The first-stage coefficient estimate was −0.003 (*P* < .001), and the *F* statistic was 450.73.

Differential distance to the nearest MA-specialized HHA met the criterion of a strong instrument,^20^ as a 1-mile increase in differential distance was associated with a lower likelihood of admission to a more MA-specialized HHA (0.3 percentage points; SE, 0.015 percentage points; *F* statistic, 450.73). This association was confirmed visually as shown in Figure 2, which depicts the first stage and shows that the probability of receiving care from a more MA-specialized HHA was lower as the differential distance to the nearest MA-specialized HHA increased.

In our 2-stage least-squares model, we identified no difference in the hospitalization rate during an HHA episode among patients receiving care from more MA-specialized HHAs (coefficient [SE], 0.006 [0.009]; *P* = .70) but slightly higher hospitalizations after discharge from the HHA at both 30 days (coefficient [SE], 0.021 [0.008]; *P* = .008) and 90 days (coefficient [SE], 0.041 [0.013]; *P* = .002). Receiving care from more MA-specialized HHAs was also associated with a 15.14-day shorter length of stay (SE, 2.84; *P* < .001) and 9.40 fewer total visits received during the episode (SE, 1.15; *P* < .001) (Table 2). We also found that the associated reduction in visits was greater for the combined measure of occupational therapy, physical therapy, and speech-language pathology visits (coefficient [SE], −4.74 [0.68]; *P* < .001) and skilled nursing visits (coefficient [SE], −3.90 [0.56]; *P* < .001) compared with medical social services and home health aide visits (coefficient [SE], −0.53 [0.16]; *P* = .001]) (eTable 1 in Supplement 1). We observed no difference in mortality and slightly higher nursing home admissions more than 90 days after discharge from an HHA. Estimates from a naive approach using ordinary least squares generated different results, including lower hospitalizations during the HHA episode (coefficient [SE], −0.026 [0.004]; *P* < .001) and estimates that were smaller in magnitude for the reduction in length of stay and total visits (Table 2). These findings suggest evidence of patient-level selection bias and validate the need for an instrumental variable approach.

**Table 2.** Ordinary Least-Squares and Instrumental Variable Analysis Results^a^

Variable	Ordinary least-squares analysis		Instrumental variable analysis	
	Coefficient (SE)	*P* value	Coefficient (SE)	*P* value
Hospitalization during HHA episode	−0.026 (0.004)	<.001	0.004 (0.009)	.70
Hospitalizations after discharge				
30 d	−0.001 (0.003)	.74	0.021 (0.008)	.008
90 d	−0.004 (0.005)	.47	0.041 (0.013)	.002
Length of stay, d	−12.97 (1.37)	<.001	−15.14 (2.84)	<.001
Total visits, No.	−5.76 (0.53)	<.001	−9.40 (1.15)	<.001

Abbreviation: HHA, home health agency.

^a^ All regression models included the patient and HHA characteristics listed in Table 1, as well as county fixed effects and SEs clustered at the HHA level.

Among a subgroup of postacute MA patients who were recently discharged from the hospital prior to initiating HHA care, we identified no difference in hospitalizations during HHA care or after discharge from the HHA. The estimates for the shorter length of stay (coefficient [SE], −14.75 [2.90] days; *P* < .001) and fewer number of total visits (coefficient [SE], −10.24 [1.29] visits; *P* < .001) were consistent with the main sample in magnitude and direction (eTable 2 in Supplement 1).

We also tested the strength of our instrument by limiting the sample to patients who received care from an HHA that was located far from their primary residence. In these first-stage regressions, the coefficients and *F* statistics were substantially smaller compared with the overall sample (eTable 3 in Supplement 1) and support the assumption that individuals receive care from more MA-specialized HHAs because of proximity and not because of other, unobserved patient-level characteristics that may be associated with being located closer to an MA-specialized HHA. Finally, we found balance among patient characteristics between these 2 groups, suggesting no obvious association between differential distance and observable patient characteristics (eTable 4 in Supplement 1).

Our results were also consistent in model specifications without patient- or HHA-level controls, specifications with patient-level controls but not HHA-level controls (eTable 5 in Supplement 1), specifications with standard errors clustered at the county level instead of the HHA level (eTable 6 in Supplement 1), and specifications with a binary measure of MA-specialization instead of a continuous measure (eTable 7 in Supplement 1). Finally, we observed evidence of a dose-response association because defining MA specialization based on the 90th percentile instead of the 75th percentile of the HHA-level share of MA patients was associated with larger differences in our outcome measures (eTable 7 in Supplement 1).

## Discussion

In this cohort study of MA patients, care received from more MA-specialized HHAs was associated with shorter lengths of stay and fewer total visits. We also observed slightly higher hospitalizations after discharge from the HHA and nursing home admissions more than 90 days after HHA discharge but no difference in the likelihood of becoming a long-stay nursing home resident or increased mortality. Given the potential administrative hurdles associated with being part of MA plans’ narrow networks and obtaining authorization for the number of days and visits covered, it is unsurprising that some HHAs specialize in delivering care for MA patients while others have little experience (>25% of HHAs in 2019 reported caring for no MA patients).

Our study differs from previous literature comparing the use of home health between MA and traditional Medicare,^5,6,9,10^ which was often limited due to baseline differences in demographic and clinical characteristics. We addressed limitations from these prior studies by taking a different perspective and exclusively studying MA patients who received HHA care. In particular, we estimated outcomes for the compliers (ie, the marginal patients who received care from an MA-specialized HHA due to their relative proximity), and our findings were conditional on patient demographic and clinical characteristics to account for potential differences in the selection of an HHA.

First, we identified lower use of health care services during the HHA episode, with fewer total visits and visits of each type. Building on prior work finding that MA patients already receive fewer HHA visits and shorter stays compared with traditional Medicare patients,^9^ our results suggest heterogeneity in the use of services among MA patients by HHA MA specialization and may magnify the existing differences in care between MA and traditional Medicare HHA patients.

Second, although we did not observe a statistically significant difference in hospitalizations during the HHA episode, we did find more hospitalizations after discharge and nursing home admissions. It is possible that MA plans covering patient care in MA-specialized HHAs have stricter caps on the services authorized or MA patients in MA-specialized HHAs may meet the goals outlined in their plan of care more quickly, such that they are discharged earlier and with fewer services. However, these patients may have greater long-term needs that result in the need for additional acute services, such as a hospitalization or short-term nursing home care.

Finally, our work is timely and important given the expected continued growth of enrollment in MA and signals by the Trump administration to increase enrollment in managed care plans, such as MA.^3^ Our results suggest that HHAs with greater exposure to MA patients provide fewer services and for shorter periods compared with other HHAs with less exposure to MA patients. Such a reduction in services would be a concern if it was accompanied by an increase in other adverse outcomes, such as hospitalizations during the HHA episode or mortality. Because this study identifies higher use of acute care after HHA discharge, it will be important to monitor how fewer HHA services among MA patients fit into the broader acute care landscape. It will also be important to continue to monitor these longer-term outcomes in the Centers for Medicare & Medicaid Services Care Compare quality data.

### Limitations

This study has several limitations. First, our study is based on data prior to the COVID-19 pandemic and the 2020 implementation of the Patient-Driven Groupings Model, which bases payment on patient clinical characteristics rather than on the volume of services provided. As a result, the incentives for HHAs to deliver care under the Patient-Driven Groupings Model and experience of MA beneficiaries receiving care from MA-specialized HHAs may be different today from our 2019 results. Second, because our sample was limited to MA beneficiaries, these results do not measure the influence of receiving care from an MA-specialized HHA for traditional Medicare beneficiaries, which might vary due to differences in demographic and clinical characteristics. Third, our patient-level instrumental variable did not address endogeneity at the HHA level. Thus, the findings may be driven by other, unmeasured HHA characteristics associated with more MA specialization. Similarly, there may be reverse causality as an HHA’s willingness to comply with MA’s restrictions on service use may have led it to obtain more MA patient referrals. Fourth, we excluded patients who had an OASIS record but were missing corresponding HHA claims (eTable 8 in Supplement 1). As a result, there may be potential bias due to missing HHA claims that might be associated with HHA characteristics. Finally, we calculated differential distance from the patient’s zip code to geocodes for the HHA home office. Using the HHA home office may be an imperfect measure of proximity to the nearest HHA, as HHA staff travel to patients’ homes to deliver care.

## Conclusions

In this cohort study of MA patients whose choice of receiving care from an HHA with more or less exposure to MA patients was driven by the relative proximity of the HHA, we found that receiving care from more MA-specialized HHAs was associated with shorter stays and fewer visits alongside slightly higher hospitalizations after discharge from the HHA. Our results suggest that receiving care from HHAs with greater exposure to MA plans was associated with lower health care service use during the HHA episode, without negative outcomes such as mortality, but had unclear implications for care use after discharge from the HHA. These findings are important given the costs associated with delivering home health care and the expected growth of MA enrollment in the coming years.

## Notes

Supplement 1. **eFigure.** Study Flowchart **eTable 1.** Instrumental Variable Results for Supplemental Outcomes **eTable 2.** Instrumental Variable Results for Postacute Care Subgroup **eTable 3.** First-Stage Results for Vacationers **eTable 4.** Characteristics by Differential Distance **eTable 5.** Sensitivity Analysis **eTable 6.** Alternative Instrumental Variable Analysis With Binary and Continuous Share Medicare Advantage (75th and 90th Percentile), Standard Errors Clustered at County Level **eTable 7.** Alternative Instrumental Variable Analysis With Binary and Continuous Share Medicare Advantage (75th and 90th Percentile), Standard Errors Clustered at HHA Level **eTable 8.** Comparison of Home Health Agency Characteristics by Match to OASIS and Encounter Claims

Supplement 2.
Data Sharing Statement
