# Supplementary material for: Specialization of Home Health Agencies to Deliver Care for Medicare Advantage Patients
Source: JAMA Netw Open. 2025 Aug 4;8(8):e2525336. doi: 10.1001/jamanetworkopen.2025.25336 (PMC12322794; doi:10.1001/jamanetworkopen.2025.25336)
Supplement: Supplement 1. — eFigure. Study Flowchart eTable 1. Instrumental Variable Results for Supplemental Outcomes eTable 2. Instrumental Variable Results for Postacute Care Subgroup eTable 3. First-Stage Results for Vacationers eTable 4. Characteristics by Differential Distance eTable 5. Sensitivity Analysis eTable 6. Alternative Instrumental Variable Analysis With Binary and Continuous Share Medicare Advantage (75th and 90th Percentile), Standard Errors Clustered at County Level eTable 7. Alternative Instrumental Variable Analysis With Binary and Continuous Share Medicare Advantage (75th and 90th Percentile), Standard Errors Clustered at HHA Level eTable 8. Comparison of Home Health Agency Characteristics by Match to OASIS and Encounter Claims [file jamanetwopen-e2525336-s001.pdf]

Supplemental Online Content

Chen AC, Fu CX, Grabowski DC. Specialization of home health agencies to deliver care for Medicare Advantage patients. *JAMA Netw Open*. 2025;8(8):e2525336. doi:10.1001/jamanetworkopen.2025.25336

eFigure. Study Flowchart

eTable 1. Instrumental Variable Results for Supplemental Outcomes

eTable 2. Instrumental Variable Results for Postacute Care Subgroup

eTable 3. First-Stage Results for Vacationers

eTable 4. Characteristics by Differential Distance

eTable 5. Sensitivity Analysis

eTable 6. Alternative Instrumental Variable Analysis With Binary and Continuous Share Medicare Advantage (75th and 90th Percentile), Standard Errors Clustered at County Level

eTable 7. Alternative Instrumental Variable Analysis With Binary and Continuous Share Medicare Advantage (75th and 90th Percentile), Standard Errors Clustered at HHA Level

eTable 8. Comparison of Home Health Agency Characteristics by Match to OASIS and Encounter Claims

This supplemental material has been provided by the authors to give readers additional information about their work.

© 2025 Chen AC et al. *JAMA Network Open*.

eFigure. Study Flowchart

Unique Medicare patients who received home health agency (HHA) care in 2019, based on OASIS
N= 5,281,705

Keep patients with start of HHA care date in 2019
N=4,191,752

Keep patients enrolled in Medicare Advantage (MA) only
N=1,425,025

Keep MA patients continously enrolled in 2019
N=1,300,176

Keep MA patients residing in contiguous 48 states
N=1,279,613

Drop patients who had an OASIS claim in 2018
N=941,689

Drop patients without HHA MA encounter claims
N=749,719

NOTES. OASIS is Outcome and Assessment Information Set.

© 2025 Chen AC et al. *JAMA Network Open*.

eTable 1. Instrumental Variable Results for Supplemental Outcomes

	Coefficient	SE	p-value
**Number of Visits**			
Skilled Nursing	-3.902	0.560	<0.001
Therapy (OT, PT, SLP)	-4.743	0.681	<0.001
Medical Social Services and Home Health Aide	-0.532	0.160	0.001
**Mortality from HHA discharge date**			
30-day	-0.003	0.002	0.05
90-day	-0.002	0.002	0.33
180-day	-0.003	0.002	0.21
**Nursing Home Admission from HHA discharge date**			
Admission from HHA discharge date			
90-day	0.019	0.009	0.04
180-day	0.029	0.012	0.01
365-day	0.057	0.016	<0.001
Long-Stay Status			
180-day	-0.004	0.005	0.44
365-day	-0.003	0.006	0.65

NOTES. SE is standard error; HHA is home health agency; OT is occupational therapy; PT is physical therapy; SLP is speech language pathology. All regression models include the patient and HHA characteristics listed in Table 1 as well as county fixed effects and robust standard errors clustered at the HHA-level. Long-stay status was determined based on MDS target and admission dates that were at least 90-days apart.

© 2025 Chen AC et al. *JAMA Network Open*.

eTable 2. Instrumental Variable Results for Postacute Care Subgroup

	Coefficient	SE	p-value	F-stat
**First Stage**	-0.003	0.0002	<0.001	384.72
**Outcomes**				
Hospitalization during HHA episode	-0.005	0.01	0.64	
Hospitalizations post-discharge				
30-day	0.003	0.01	0.79	
90-day	0.03	0.02	0.06	
Length of Stay	-14.75	2.90	<0.001	
Total Visits	-10.24	1.29	<0.001	

NOTES. SE is standard error; HHA is home health agency. All regression models include the patient and HHA characteristics listed in Table 1 as well as county fixed effects and robust standard errors clustered at the HHA-level.

© 2025 Chen AC et al. *JAMA Network Open*.

eTable 3. First-Stage Results for Vacationers

	Overall	50+ miles	100+ miles	150+ miles
Coefficient	-0.003	-0.001	-0.0007	-0.0005
F-statistic	450.73	44.79	5.46	3.51
N	749,719	54,758	20,871	14,841
p-value	p<0.001	p<0.001	p<0.001	0.06

NOTES. All regression models include the patient and HHA characteristics listed in Table 1 as well as county fixed effects and robust standard errors clustered at the HHA-level. Vacationer sample includes patients who received care from a home health agency that was located 50+, 100+ or 150+ miles away from the ZIP code of their primary residence.

© 2025 Chen AC et al. *JAMA Network Open*.

eTable 4. Characteristics by Differential Distance

	IV <= county-level median IV	IV > county level median IV
**N (%)**	383,151 (51.11%)	366,568 (48.89%)
**Variable**	**Mean**	**Mean**
**Patient Characteristics**		
Age	76.17	76.14
Sex (male)	38.10%	38.72%
Race		
Black	14.41%	14.77%
Hispanic	7.92%	8.23%
White	74.62%	73.98%
Other Race	2.39%	2.37%
Dual-Eligible	26.99%	25.49%
Post-Acute	36.30%	36.26%
Prior Functioning (Cognitive Status)		
Dependent	2.67%	2.70%
Needed Some Help	15.75%	16.03%
Independent	30.27%	30.34%
Missing	51.19%	50.81%
Unknown	0.11%	0.12%
Prior Functioning (Mobility)		
Dependent	1.31%	1.35%
Needed Some Help	15.82%	16.47%
Independent	31.32%	31.03%
Missing	51.20%	50.82%
Unknown	0.35%	0.34%
Prior Functioning (Self-care)		
Dependent	1.56%	1.63%
Needed Some Help	17.32%	17.86%
Independent	29.88%	29.67%
Missing	51.19%	50.81%
Unknown	0.05%	0.05%
Prior Functioning (Stairs)		
Dependent	2.33%	2.39%
Needed Some Help	16.60%	16.91%
Independent	24.35%	24.07%
Missing	51.46%	51.10%
Unknown	5.26%	5.52%
Primary Diagnosis Severity Rating		
Asymptomatic, no treatment needed at this time.	0.01%	0.01%
Symptoms well controlled with current therapy	0.67%	0.61%

© 2025 Chen AC et al. *JAMA Network Open*.

Symptoms controlled with difficulty, affecting daily functioning	13.36%	13.72%
Symptoms poorly controlled, patient needs frequent adjustment in treatment and dose monitoring	24.92%	25.24%
Symptoms poorly controlled, history of rehospitalizations	3.38%	3.10%
Missing	57.66%	57.33%
**HHA characteristics**		
Ownership		
Non-profit	28.37%	28.56%
For-profit	52.14%	56.17%
Government	19.49%	15.27%
Star Rating	3.50	3.52
Urban	66.66%	64.72%
Suburban	32.66%	33.92%
Rural	0.69%	1.36%
Annual Number of Patients	613.10	507.92
**Outcomes**		
Hospitalization during HHA episode	7.82%	7.88%
Hospitalizations post-discharge		
30-day	7.80%	7.92%
90-day	15.33%	15.53%
Length of Stay	37.78	38.43
Total Visits	10.60	10.98

NOTES. IV is instrumental variable; HHA is home health agency. Other race also includes Asian, North American Native, and unknown race.

© 2025 Chen AC et al. *JAMA Network Open*.

eTable 5. Sensitivity Analysis

	(1) No characteristics			(2) Patient characteristics		
	Coefficient	SE	p-value	Coefficient	SE	p-value
Hospitalization during HHA episode	-0.008	0.02	0.69	0.003	0.02	0.85
Hospitalizations post-discharge						
30-day	-0.05	0.01	0.002	-0.04	0.01	0.003
90-day	-0.05	0.02	0.01	-0.05	0.02	0.02
Length of Stay	-11.49	6.84	0.09	-10.58	6.18	0.09
Total Visits	-6.06	2.11	0.004	-8.84	2.06	0.005

NOTES. SE is standard error; HHA is home health agency. No characteristics model includes county fixed effects only. Patient characteristics model includes the patient characteristics listed in Table 1 and county fixed effects. Robust standard clustered at the HHA-level.

© 2025 Chen AC et al. *JAMA Network Open*.

eTable 6. Alternative Instrumental Variable Analysis With Binary and Continuous Share Medicare Advantage (75th and 90th percentile), Standard Errors Clustered at County Level

**Treatment**	**Binary Share MA**						**Continuous Share MA**					
**Differential Distance**	**Based on 75th percentile**			**Based on 90th percentile**			**Based on 75th percentile**			**Based on 90h percentile**		
**First Stage** (coefficient; p-value; F-stat)	-0.001 (p<0.001); 414.77			-0.004 (p<0.001); 96.58			-0.003 (p<0.001); 354.26			-0.002 (p<0.001); 90.38		
	**Coefficient**	**SE**	**p-value**	**Coefficient**	**SE**	**p-value**	**Coefficient**	**SE**	**p-value**	**Coefficient**	**SE**	**p-value**
Hospitalization during HHA episode	0.001	0.004	0.76	-0.022	0.005	<0.001	0.004	0.012	0.76	-0.045	0.011	<0.001
**Hospitalizations post-discharge**												
30-day	0.007	0.003	0.01	0.030	0.005	<0.001	0.021	0.008	0.008	0.061	0.009	<0.001
90-day	0.013	0.005	0.005	0.050	0.008	<0.001	0.041	0.014	0.004	0.104	0.016	<0.001
Length of Stay	-4.906	0.956	<0.001	-17.420	1.841	<0.001	-15.144	3.045	<0.001	-36.075	3.599	<0.001
Total Visits	-3.045	0.363	<0.001	-8.149	0.733	<0.001	-9.399	0.966	<0.001	-16.875	1.555	<0.001
**Number of Visits**												
Skilled Nursing	-1.264	0.182	<0.001	-3.295	0.328	<0.001	-3.902	0.516	<0.001	-6.825	0.687	<0.001
Therapy (OT, PT, SLP)	-1.537	0.207	<0.001	-4.297	0.471	<0.001	-4.743	0.564	<0.001	-8.898	0.979	<0.001
Medical Social Services and Home Health Aide	-0.172	0.049	0.001	-0.521	0.119	<0.001	-0.532	0.154	0.001	-1.079	0.255	<0.001
**Mortality from HHA discharge date**												
30-day	-0.001	0.000	0.02	-0.001	0.001	0.09	-0.003	0.001	0.02	-0.003	0.002	0.09
90-day	-0.001	0.001	0.32	-0.002	0.001	0.09	-0.002	0.002	0.32	-0.003	0.002	0.09
180-day	-0.001	0.001	0.22	-0.002	0.001	0.03	-0.003	0.002	0.22	-0.005	0.002	0.03
**Nursing Home Admission from HHA discharge date** ***Admission from HHA discharge date***												
90-day	0.006	0.003	0.02	0.026	0.006	<0.001	0.019	0.008	0.02	0.054	0.012	<0.001
180-day	0.009	0.003	0.006	0.036	0.008	<0.001	0.029	0.010	0.005	0.074	0.016	<0.001

© 2025 Chen AC et al. *JAMA Network Open*.

365-day	0.019	0.005	<0.001	0.057	0.010	<0.001	0.057	0.014	<0.001	0.119	0.020	<0.001
Long-Stay Status												
180-day	-0.001	0.002	0.47	-0.001	0.002	0.59	-0.004	0.005	0.47	-0.003	0.005	0.59
365-day	-0.001	0.002	0.70	-0.002	0.003	0.60	-0.003	0.007	0.70	-0.004	0.007	0.60

NOTES. MA is Medicare Advantage; SE is standard error; HHA is home health agency; OT is occupational therapy; PT is physical therapy; SLP is speech language pathology. All regression models include the patient and HHA characteristics listed in Table 1 as well as county fixed effects and robust standard errors clustered at the county-level.

© 2025 Chen AC et al. *JAMA Network Open*.

eTable 7. Alternative Instrumental Variable Analysis With Binary and Continuous Share Medicare Advantage (75th and 90th Percentile), Standard Errors Clustered at HHA Level

**Treatment**	**Binary Share MA**						**Continuous Share MA**					
**Differential Distance**	**Based on 75th percentile**			**Based on 90th percentile**			**Based on 75th percentile**			**Based on 90th percentile**		
**First Stage** (coefficient; p-value; F-stat)	-0.01 (p<0.001); 650.35			-.004 (p<0.001) 198.49			-.003 (p<0.001) 450.73			-.002 (p<0.001) 196.18		
	**Coefficient**	**SE**	**p-value**	**Coefficient**	**SE**	**p-value**	**Coefficient**	**SE**	**p-value**	**Coefficient**	**SE**	**p-value**
Hospitalization during HHA episode	0.001	0.003	0.70	-0.022	0.005	<0.001	0.004	0.009	0.70	-0.045	0.010	<0.001
**Hospitalizations post-discharge**												
30-day	0.007	0.003	0.008	0.030	0.005	<0.001	0.021	0.008	0.008	0.061	0.009	<0.001
90-day	0.013	0.004	0.002	0.050	0.007	<0.001	0.041	0.013	0.002	0.104	0.015	<0.001
Length of Stay	-4.906	0.905	<0.001	-17.420	1.611	<0.001	-15.144	2.839	<0.001	-36.075	3.182	<0.001
Total Visits	-3.045	0.386	<0.001	-8.149	0.652	<0.001	-9.399	1.145	<0.001	-16.875	1.330	<0.001
**Number of Visits**												
Skilled Nursing	-1.264	0.183	<0.001	-3.295	0.309	<0.001	-3.902	0.560	<0.001	-6.825	0.645	<0.001
Therapy (OT, PT, SLP)	-1.537	0.229	<0.001	-4.297	0.397	<0.001	-4.743	0.681	<0.001	-8.898	0.801	<0.001
Medical Social Services and Home Health Aide	-0.172	0.051	0.001	-0.521	0.097	<0.001	-0.532	0.160	0.001	-1.079	0.208	<0.001
**Mortality from HHA discharge date**												
30-day	-0.001	0.001	0.05	-0.001	0.001	0.08	-0.003	0.002	0.05	-0.003	0.002	0.08
90-day	-0.001	0.001	0.33	-0.002	0.001	0.10	-0.002	0.002	0.33	-0.003	0.002	0.10
180-day	-0.001	0.001	0.21	-0.002	0.001	0.04	-0.003	0.002	0.21	-0.005	0.002	0.04
**Nursing Home Admission from HHA discharge date**												
Admission from HHA discharge date												
90-day	0.006	0.003	0.03	0.026	0.005	<0.001	0.019	0.009	0.04	0.054	0.009	<0.001
180-day	0.009	0.004	0.01	0.036	0.006	<0.001	0.029	0.012	0.01	0.074	0.012	<0.001

© 2025 Chen AC et al. *JAMA Network Open*.

365-day	0.019	0.005	<0.001	0.057	0.008	<0.001	0.057	0.016	<0.001	0.119	0.017	<0.001
Long-Stay Status												
180-day	-0.001	0.002	0.44	-0.001	0.002	0.58	-0.004	0.005	0.44	-0.003	0.005	0.58
365-day	-0.001	0.002	0.65	-0.002	0.003	0.54	-0.003	0.006	0.65	-0.004	0.006	0.54

NOTES. MA is Medicare Advantage; SE is standard error; HHA is home health agency; OT is occupational therapy; PT is physical therapy; SLP is speech language pathology. All regression models include the patient and HHA characteristics listed in Table 1 as well as county fixed effects and robust standard errors clustered at the HHA-level.

© 2025 Chen AC et al. *JAMA Network Open*.

eTable 8. Comparison of Home Health Agency Characteristics by Match to OASIS and Encounter Claims

	MA beneficiaries identified in OASIS	MA beneficiaries identified in OASIS and HHA encounter claims	MA beneficiaries identified in OASIS but not HHA encounter claims
N (percent)	925,685	749,719 (80.99%)	175,966 (19.01%)
**Home Health Agency Characteristics**			
Ownership Type (percent)			
Non-Profit	27.66%	28.46%	24.26%
For-Profit	55.26%	54.11%	60.17%
Government	17.07%	17.43%	15.57%
Quality star ratings (mean)	3.50	3.51	3.47
Urban	65.02%	65.71%	62.09%
Suburban	34.03%	33.28%	37.23%
Rural	0.95%	1.01%	0.67%
Total patients per HHA	559.64	561.67	550.97

NOTES. HHA is home health agency.

© 2025 Chen AC et al. *JAMA Network Open*.
