# Supplementary material for: Specialization of Home Health Agencies to Deliver Care for Medicare Advantage Patients
Source: JAMA Netw Open. 2025 Aug 4;8(8):e2525336. doi: 10.1001/jamanetworkopen.2025.25336 (PMC12322794; doi:10.1001/jamanetworkopen.2025.25336)
Supplement: Supplement 2. — Data Sharing Statement [file jamanetwopen-e2525336-s002.pdf]

Data Sharing Statement

Chen. Specialization of Home Health Agencies to Deliver Care for Medicare Advantage Patients. *JAMA Netw Open*. Published August 04, 2025.
doi:10.1001/jamanetworkopen.2025.25336

Data
Data available: No
